# Resistant xylem from roots to peduncles sustains reproductive water supply after drought-induced cavitation of wheat leaves

**DOI:** 10.1093/aob/mcad048

**Published:** 2023-03-22

**Authors:** Beatrice L Harrison Day, Timothy J Brodribb

**Affiliations:** School of Natural Sciences, University of Tasmania, Private Bag 55, Hobart, TAS 7001, Australia; School of Natural Sciences, University of Tasmania, Private Bag 55, Hobart, TAS 7001, Australia

**Keywords:** Drought, optical vulnerability method, rehydration, roots, segmentation, *Triticum aestivum*, wheat, xylem cavitation

## Abstract

**Background and Aims:**

Many annual grasses exhibit drought-avoiding life cycles in which rapid reproduction must be completed before soil water is exhausted. This strategy would seem to require a hydraulic system capable of sustaining reproduction at all costs to the rest of the plant, yet little is known about the whole-plant structure of hydraulic vulnerability in grasses.

**Methods:**

We examine vulnerability to water-stress-induced xylem cavitation in roots, flag leaves, and basal and apical regions of peduncles of wheat (*Triticum aestivum* L. ‘Krichauff’) to understand the staged failure of xylem function in severe drought. The functionality of segmented vulnerabilities is tested by conducting rehydration experiments after acute dehydration.

**Key Results:**

We show that water supply to peduncles is more drought resistant than in leaves due to greater xylem cavitation resistance, ensuring a pathway of water can be maintained from the roots to the reproductive tissues even after severe water deficit. Differential rehydration of peduncles compared to leaves following drought confirmed the functionality of xylem supply from roots to seed after water stress sufficient to completely cavitate flag leaf vessels.

**Conclusions:**

These results demonstrate that a proportion of the hydraulic pathway between roots and seeds remains functional under extreme dehydration, suggesting that vulnerability traits in this key grass species reflect its reproductive strategy.

## INTRODUCTION

Monocot plants dominate global landscapes, making up almost a quarter of all angiosperm species (Soltis and Soltis, 2004). Of these, annual grasses represent a substantial proportion of native and crop species around the world, often growing in unpredictable water-limited environments where rapid reproduction is essential (Hahn *et al.*, 2021). Extensive work has described the reproductive ecology of grass species in water-limited environments, where they are often categorized through drought avoidance and tolerance traits (Araus *et al.*, 2002; Sherrard and Maherali, 2006; Volaire *et al.*, 2014; Volaire, 2018; Garbowski *et al.*, 2020). Yet there is little physiological information about how the plant vascular system in such species has evolved to enable reproductive traits during drought.

Hydraulic failure through xylem cavitation is a causal factor determining plant survival under water stress (Choat *et al.*, 2018; Barigah *et al.*, 2013; Johnson *et al.*, 2018; Brodribb *et al.*, 2021). One informative way of characterizing plant damage under drought is to examine the sequence of xylem cavitation between tissues within a plant. The hydraulic vulnerability segmentation hypothesis uses patterns of tissue preservation and sacrifice to consider the carbon investment and cost of losing tissues under drought (Tyree and Ewers, 1991; Tsuda and Tyree, 1997; Choat *et al.*, 2005; Hochberg *et al.*, 2016). The theory suggests more long-lived non-redundant structures such as stems that require large carbon investments should be more resistant to embolism and persist longer under water stress than short-lived distal tissues such as leaves (Cochard *et al.*, 1992; Zimmermann, 2013; Pivovaroff *et al.*, 2014; Nolf *et al.*, 2015; Charrier *et al.*, 2016, Johnson *et al.*, 2016). This theory was developed to examine the variation in vulnerability between leaves and stems but may be applied more broadly across whole plants. Beyond predicting species mortality, varying cavitation vulnerabilities within plants reveal how changes in the functionality of xylem among tissues may affect the distribution of water resources (Bourbia *et al.*, 2020; Harrison Day *et al.*, 2021).

Traditionally, hydraulic vulnerability segmentation has examined the differences in vulnerability to cavitation between leaves and subtending tissues of woody species where transpirationally costly leaves are isolated early under water stress through cavitation, thus extending the survival of the individual plant (Tyree and Ewers, 1991; Tyree *et al.*, 1993; Johnson *et al.*, 2011; Pivovaroff *et al.*, 2014; Klepsch *et al.*, 2018; Levionnois *et al.*, 2020). In these long-lived woody plants, leaf sacrifice to preserve water resources presents a clear ecophysiological advantage to a plant that must survive successive dry spells, persisting long enough to regrow leaves and resume function under more favourable conditions. In these perennials, flowers also represent a transpirationally costly tissue that may also be lost early during drought through cavitation to prolong plant survival (Zhang and Brodribb, 2017; Fernandes *et al.*, 2018; Bourbia *et al.*, 2020). Understanding when flowers and fruit are sacrificed relative to leaves and stems allows us to examine the reproductive fitness of different plant groups under drought. Consequently, the staged failure of tissues through cavitation may be examined in terms of lasting survival and fitness strategy as well as carbon costs (Lens *et al.*, 2013; Rodriguez-Dominguez *et al.*, 2018; Dória *et al.*, 2018; Bourbia *et al.*, 2020; Harrison Day *et al.*, 2021).

This picture of hydraulic vulnerability segmentation between vulnerable leaves and flowers and tough stems is optimized for the ecophysiology of long-lived plants (Zhang and Brodribb, 2017; Fernandes *et al.*, 2018; Bourbia *et al.*, 2020). The hydraulic vulnerability pattern in annual grass species is likely to be very different to that in woody and perennial plants, but little is known about whole plant hydraulic vulnerability traits in this group. Annual grass species, many crops included, commonly grow vegetatively in spring and become reproductive during warmer summer periods when water deficit is likely to be routine (Araus *et al.*, 2002; Volaire, 2003; Hahn *et al.*, 2021). The hydraulic strategy and allocation of water resources during drought is likely to reflect the ecophysiological traits of annual species that will not live for successive seasons but have the potential to complete reproduction given the right allocation of resources and combination of hydraulic traits. The strategy of flower shedding under drought as seen in perennial plants (Bourbia *et al.*, 2020) is counterproductive in annual plants where rapid reproduction is essential. Vulnerability experiments in domesticated annual tomato suggest that water supply to reproductive tissue is prioritized above leaves during drought through greater resistance to cavitation (Harrison Day *et al.*, 2021). In these annual plants, early leaf sacrifice slows the decline of xylem water potential (Gleason *et al.*, 2014), ensuring the prolonged delivery of water to reproductive tissues even under acute drying, supported by a cavitation-resistant pathway of xylem supplying the fruit (Harrison Day *et al.*, 2021). This raises the question of whether annual grasses, a key plant group with significant relevance to crops and wild species, also show greater investment in reproduction, and thus increased cavitation resistance in reproductive water supply, over transient vegetative tissues that will die at the end of the growing season regardless of drought stress.

Although various studies have compared different parts of the water transport system within individual plants, there has been no attempt thus far to examine the vulnerability properties of all major components of the vascular system from roots to reproductive tissues simultaneously *in situ* in a grass species. A fundamental constraint on water transport and the availability of water resources to the plant is the vulnerability of root networks to xylem cavitation. Root vulnerabilities have been estimated using diverse techniques with varying levels of invasiveness (Sperry and Saliendra, 1994; Alder *et al.*, 1997; Sperry and Ikeda, 1997; Kavanagh *et al.*, 1999; Pratt *et al.*, 2015; Johnson *et al.*, 2016), but recent techniques such as the Optical Method and micro-computed tomography (micro-CT) have allowed xylem cavitation to be quantified non-invasively in roots (Skelton *et al.*, 2017; Rodriguez-Dominguez *et al.*, 2018). These new techniques provide a new opportunity to explore the relationship between xylem vulnerability segmentation and plant reproductive strategy from a whole-plant perspective by examining many tissues concurrently in undamaged plants. Including roots in this picture provides the key link between the soil and above-ground tissues, determining when the plant is isolated from soil resources (Rodriguez-Dominguez *et al.*, 2018), although the complete pathway of water to reproductive tissues remains unstudied. Here we examine the entire water transport pathway in wheat (*Triticum aestivum* L. ‘Krichauff’) including roots, leaves and peduncles. We endeavour to understand how tissue-specific hydraulic vulnerability traits in this key monocot crop species intersect with plant strategy and reproductive fitness under soil drying. We hypothesize, considering its annual status, that water supply to the reproductive tissues in wheat should show greater resistance to xylem cavitation under severe water stress than leaves. Specifically, the xylem pathway from the root through to the peduncle, the vascular conduit to the seed, would be expected to show greater resistance to cavitation than the evaporatively costly leaves under water stress.

Although tests of xylem cavitation have been commonly used to compare tissue and species vulnerability to drought, the direct mechanistic link between cavitation and tissue death has been difficult to prove (Brodribb *et al.*, 2021). Similarly, the link between tissue cavitation, hydraulic vulnerability segmentation and plant ecophysiological strategy remains tenuous without a functional test of whether the xylem pathways predicted to be intact during drought according to xylem vulnerability measurements are indeed functional upon rehydration. In addition to quantifying the vulnerability of different vascular tissues to cavitation, we use rehydration experiments to directly test whether the differential vulnerability of tissues corresponds to prioritized water supply during water stress and recovery. Thus, we present a detailed whole-plant investigation into the hydraulic dynamics of wheat tissues, examining the functional implications of xylem priority towards preserving water supply from roots to seeds in wheat.

## MATERIALS AND METHODS

### Plant material and preparation

Bread wheat (*T. aestivum* L. Krichauff) was grown in glasshouse facilities at the University of Tasmania, Australia. Seeds were germinated directly in 2-L pots filled with potting mix (7 : 4 mix of composted fine pine bark and coarse washed river sand) and a slow-release general Osmocote®. Wheat was chosen as an important grass species given its easy germination and rapid generation times, with the understanding that although selection has occurred, the hydraulic drivers of reproduction are likely to be informative. The Krichauff variety was chosen as a summer flowering cultivar with no cold vernalization requirements and with no specific drought resistance and stay-green selection. Plants were grown in average day/night temperatures of 20/15 °C under an 18-h photoperiod to ensure flowering. Daytime relative humidity was kept at ~40 % and plants were watered daily to avoid drought stress. Plant age was carefully controlled, limiting testing to the grain development phase, to mitigate confounding drought response and senescence. Plants were grown for ~8 weeks (~40 cm in height) until peduncles and inflorescences were visible and the flag leaf was fully expanded, but before mature plants showed signs of senescence (as indicated by yellowing of the spike or flag leaf). Evidence suggests that the final stage of wheat grain maturation-drying is accompanied by xylem occlusion in the peduncle, aiding the planned senescence and maturation-drying of seed, so tests were limited to young wheat plants with newly emerged reproductive spikes (Neghliz *et al.*, 2016). Experiments commenced when the plants had at least two tillers of similar developmental stage. Well-watered plants were removed from pots and their roots were very gently rinsed with water to remove most of the soil, allowing cameras to be simultaneously installed on exposed roots and above-ground tissues. Prepared plants were laid flat and allowed to dehydrate under stable lab conditions (around 30 µmol quanta m^−2^ s^−1^ of light, at 18 °C), ensuring dehydration was slow enough to maintain water potential equilibrium between tissues up until the point of isolation by cavitation but rapid enough to prevent drought-stress reproductive vigour responses or light starvation senescence, taking around 4 d to fully dry.

### Measurement of cavitation

Xylem cavitation was measured simultaneously in the leaves, roots, and basal and apical regions of the peduncle in five replicate plants to determine the order of cavitation and the relative xylem vulnerability of organs within the same plant.

Wheat plants were observed to have about three seminal roots (around 20–30 cm in length) and many (50+) lateral and fine roots branching off these seminal roots. A clean bundle of undamaged large roots (including seminal and the largest lateral roots ~0.5–1 mm in diameter) around 100 mm below the root collar were placed between two glass slides to prevent distortion and to ensure all roots were at the same focal distance from the lens. These were very gently clamped within a ‘cavicam’ (cavicam.co, Hobart), with LED white light transmitted through the root to illuminate root xylem. Images were acquired every 5 min until the whole plant was fully desiccated (roughly 4 d). These larger roots (>5 mm in diameter) were chosen because they were expected to dominate the root hydraulic conductance. Simultaneously, the middle section of flag leaf was placed between two glass slides to prevent distortion during drying and then gently clamped in a cavicam. Light was transmitted through the leaf tissue, and images were acquired every 5 min as above.

Two regions of peduncle were selected on the same tiller as the flag leaf, one directly above the flag leaf node (referred to here as basal peduncle) and one directly below the flowering spike (referred to as apical peduncle). These regions were gently wiped with damp tissue paper to remove the glaucous epicuticular layer, improving image quality. Prepared sections were clamped into individual cavicams, and images were acquired as above using reflected light. Cavitation was determined to have reached 100 % when no more pixel changes had occurred across all tissues for at least 6 h.

### Image analysis

Image stacks were analysed in ImageJ (NIH) using the optical vulnerability (OV) method (see http://www.opensourceov.org/). In brief, pixel differences between successive images indicate a change in light transmission during cavitation events as liquid water in vessels quickly transitions to air/vapour, and these may be extracted using the image subtraction method (Brodribb *et al.*, 2016). The cumulative number of cavitated pixels was assumed to account for the impact of different size embolisms on flow (Brodribb *et al.*, 2017; Gauthey *et al.*, 2020). The total embolism area per image was calculated as the summed embolized pixels for the image stack, and then cumulative pixel change (indicating area cavitated as a percentage of the total) was plotted against water potential to create vulnerability curves (Brodribb *et al.*, 2017; Bourbia *et al.*, 2020). Vulnerability curves were used to determine the water potential where 12, 50, 88 and 100 % of xylem within an individual organ had cavitated (P_12_, P_50,_ P_88_, P_100_), used as a standard to compare between organs. Rather than fitting vulnerability functions as is necessary in other techniques where many samples are used to build a single curve, the OV technique tracks embolism accumulation within individual organs, so it is most accurate to read individual values for P_12_, P_50_ and P_88_ from individual plots of cumulative embolism versus water potential.

### Root image analysis

The root vulnerability curves were carefully analysed with stringent selection criteria of large, grouped pixel clusters and ‘long’ pixel change events, and by limiting image difference events to the vascular bundle, to avoid inclusion of events other than cavitation in root image analysis. Small extra-xylary changes were excluded as non-cavitation. Slow and small changes were filtered by limiting analysis to long (>100 µm) image difference events that occurred over a single frame difference. These changes should have isolated the rapid air-filling of long xylem vessels from the slower, erratic changes likely to evolve due to phenomena such as cortical lacunae formation (Cuneo *et al.*, 2021). Although cortical lacunae formation has been shown in other species (Cuneo *et al.*, 2016, 2021), micro-CT scans of wheat show no evidence of cortical lacunae formation in this species (H. Day, unpubl. data), meaning that any long image differences limited within the xylem are likely to be xylem cavitation. These large roots contain a small quantity of extremely large central metaxylem and a larger quantity of smaller metaxylem, resulting in stepped or steep vulnerability curves that differed from the sigmoidal function usually described by the optical method (Brodribb *et al.*, 2017). The first images (~10 frames) where liquid water was visible on roots were removed from the analysis due to excessive movement as the surface water evaporated and was justified due to the knowledge that drought-induced xylem cavitation could not occur while water was present on the roots, although there was no indication of cavitation (significant pixel differences in the vascular bundle) in this initial period. In each plant, —two to six large roots per plant remained sufficiently in focus throughout the drying period to produce well-resolved cavitation sequences. A unique vulnerability curve was calculated for each individual root within a plant so that they could be examined separately as individual components of the root bundle. In addition, the roots were grouped within a replicate plant to look at mean root bundle curves per plant. To achieve this, the cumulative pixel values from the two to four roots per plant were summed to construct a cumulative vulnerability curve. Cavitation was complete after ~3 d.

### Measurement of water potential

The peduncle base was considered the best point to monitor water potential (WP) during whole plant dehydration, providing a structurally solid tissue to attach the psychrometer. The peduncle was later found to be the most resistant tissue to cavitation, allowing the longest possible measurement of WP in the plant. Psychrometers could not be placed lower on the plant (proximal to the root collar) as the basal part of the leaf surrounded the stem and could not be removed without damaging the subtending leaves. Once stomata close, the gradient of water potential between the roots and leaves collapses, meaning that psychrometer measurements from one organ may be applied reliable to all other parts of the plant until the organs are isolated through cavitation (Skelton *et al.*, 2017). As further rehydration experiments showed the recovery of water supply to peduncles during rewatering, it is highly unlikely that cavitation occurred in the intermediary stem between peduncle and root, causing the WP in the roots to differ from that shown by peduncle and leaf psychrometers. Thus, the peduncle psychrometer could be reliably applied to organs up until the point of localized runaway cavitation.

A calibrated stem psychrometer (ICT international, Armidale, Australia) was installed at the base of the peduncle immediately above the flag leaf node (Supplementary Data Fig. S1). A region of 6 mm on one side of the peduncle was gently sanded with 600 grit wet/dry sandpaper (Flexovit, Australia) to remove the cuticle. The sandpaper was chosen as an easily available fine-grit sandpaper that works under wet conditions (i.e. plant tissue). The fine grit was chosen to cause as little damage to the tissue as possible and only take off a fine layer of cuticle/epidermis. The surface was washed with distilled water to remove any debris and carefully dried. Two folded blocks of parafilm (Bemis Co. Inc., USA) (10 × 4 × 3 mm) were placed parallel on either side of the cooling chamber and the peduncle tissue was sandwiched in between these blocks to fully cover the chamber with the peduncle. This ensured no air could leak into the chamber, while providing a sturdy physical base for the psychrometer clamp, preventing the tissue from being crushed. Several layers of parafilm were then applied over the peduncle and blocks, creating an airtight seal. A square of neoprene was placed over the tissue before clamping, further protecting the tissue from crush damage. The psychrometer was set to log peduncle water potential every 15 min, with a Peltier cooling time of 5 s and a wait time of 10 s, with cooling time increased as the tissues dried. A second stem psychrometer (ICT international) was attached to the corresponding flag leaf closest to the peduncle psychrometer as a confirmation point to ensure the peduncle psychrometer was logging accurately but was not used in analyses. A 1-cm^2^ region of lamina was treated by gently sanding with 600 grit wet/dry sandpaper, washed with distilled water and carefully dried, removing the leaf cuticle. The sanded leaf surface was placed prepared side onto the chamber of a psychrometer. A square of neoprene wrapped in parafilm was placed over the leaf tissue and then clamped shut, protecting the tissue from crushing while also sealing the tissue from air. The psychrometer was set to log as above. A linear regression describing the relationship between WP and time (*r*^2^ > 0.95) was fitted to the leaf and peduncle psychrometer data set, comparing the WP measurements from organs within the same plant. The rate of decrease in WP from both organs agreed closely up until the point of leaf cavitation (Supplementary Data Fig. S2). The WP from peduncles was applied to the organs of that plant. A unique rate of WP decline was measured for each individual plant.

### Tissue rehydration following drought and cavitation

The segmented vulnerabilities established from the optical method indicated that the peduncles and supplying tissues should have the capacity to rehydrate after the point of 100 % leaf cavitation (see Results). We experimentally tested whether this differential cavitation vulnerability between roots, leaves and peduncles conferred the expected benefit to reproductive tissue in terms of functional water delivery to the seed after severe drought stress. For peduncles to rehydrate, a proportion of the root network must remain undamaged during drought stress. To test our theoretical segmented vulnerabilities, we measured the recovery of hydration in the peduncles after plants were exposed to water stress causing varying levels of cavitation in adjacent leaves.

Seven plants were grown under identical conditions and at the same developmental stage as the previous experiment. Plants were removed from pots, removing some of the soil bulk to speed up drying but leaving the root bundle relatively undisturbed in soil in order to show rehydration in root networks with no manual handling. Plants were dried under controlled laboratory conditions (around 30 µmol quanta m^−2^ s^−1^, at 18 °C). The root bundle was placed in a shallow container so that the intact root network could be submerged after drying. A psychrometer was attached to the peduncle and leaf of the same tiller (as above), measuring the decrease in WP (MPa) as the plant dried. A camera was attached to a leaf, capturing cavitation in the leaf. The seven plants were dried to increasing water stresses, resulting in staged increments of leaf and finally peduncle cavitation. The first plant was dried to −1 MPa, a water stress causing no leaf cavitation, where the plant was expected to rehydrate rapidly upon rewatering. Two plants were dried to water stress resulting in incomplete leaf cavitation, where the leaves could conceivably still rehydrate. Three plants were dried to the critical point where the leaves were fully cavitated, but the stems could still theoretically recover. This target WP was taken from the organ vulnerability (see Results) to create a water deficit at which peduncles and roots could theoretically be recovered by rewatering past the point of fatal leaf cavitation. A final plant was dried beyond the expected peduncle P_100_ WP and then rewatered to ensure no passive rehydration occurred beyond peduncle cavitation. When the target WP was reached, the intact root bundle was rewatered ensuring all root tissues were wet, and the leaves were covered with a damp paper towel. To achieve this, the shallow container containing the intact root network was filled with water, submerging the entire root bundle, and a paper towel was placed over the wet root network to slow evaporation and ensure no parts of the root bundle dried. The plants were left to rehydrate for a further 48+ h with all instruments left logging until the plant had fully rehydrated. Finally, the leaves in cameras were cut off at the end of the experiment and allowed to dry until cavitation ceased, thus providing a reference pixel area representing 100 % leaf cavitation. This reference value was used to calculate the leaf cavitation percentage at the time of rewatering. Values for recovery of leaf and peduncle psychrometer WP within an individual plant were compared to the percentage leaf cavitation at the time of rewatering.

### Statistical tests

The cavitation resistance of organs was compared using a two-way ANOVA in ‘R 1.2.5019’, examining the effect of organ and replicate on *P*_50_, including the interaction between organ and replicate. Five plants were compared, with organs treated as paired as they were tested on the same plants. This ANOVA model was tested for normality and homoscedasticity and no transformations were required. The interaction term between organ and replicate was determined to have an insignificant effect and was dropped from the model. *Post hoc* tests were determined using Tukey’s honest significant difference (HSD) test.

## RESULTS

### Hydraulic vulnerability segmentation

**Fig. 1.**
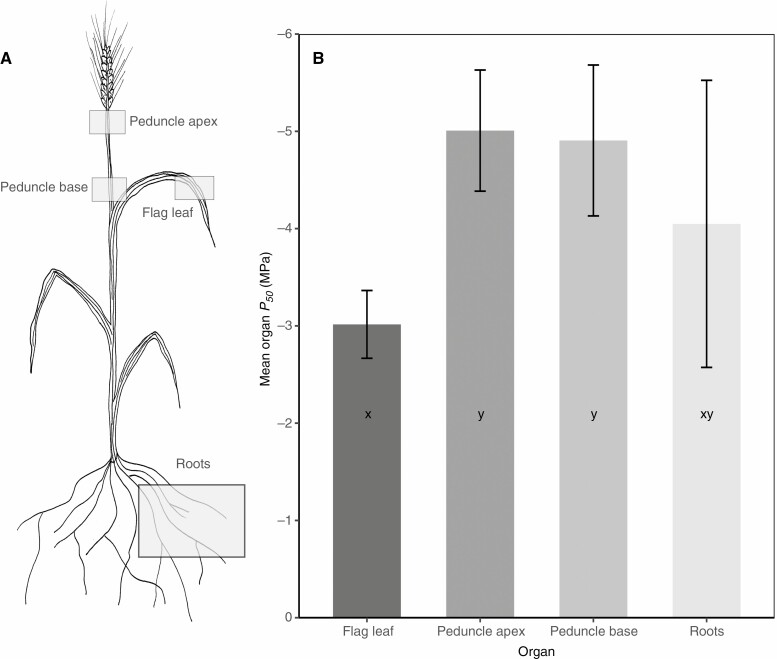
(A) Spatial diagram showing the location of bread wheat roots, flag leaves, and basal and apical regions of peduncles and (B) mean (±s.d.) *P*_50_ values from five replicates of bread wheat flag leaves, roots, and basal and apical regions of peduncles. Letters (x–z) indicate significant *post-hoc* differences between organs (*P* < 0.05).

A clear pattern of vulnerability variation emerged between tissues of the wheat plants under dehydration stress (Fig. 1). *P*_50_ values varied significantly amongst organs (*P* = 0.0001***) and replicate had a minor influence on this pattern of vulnerability (*P* = 0.046*). The flag leaves were typically the most vulnerable organ to xylem cavitation, with a mean (±s.d.) P_50_ of −3.01 ± 0.35 MPa (Fig. 1). The basal peduncle region and apical peduncle region (the lower and upper regions of the spike holding the inflorescence) were not significantly different from one another (*P* = 0.99) showing no vulnerability segmentation along the length of this key water supply to the seed (Fig. 1). Both basal and apical regions of peduncle were significantly more resistant to cavitation than leaves (*P* = 0.003 and *P* = 0.002) with mean P_50_ values of −4.90 ± 0.78 and −5.01 ± 0.62 MPa respectively. The mean *P*_50_ of roots (−4.05 MPa) was between the leaf and peduncle values, and these root values showed the greatest variation of any organs (s.d. ±1.47 MPa). Mean root P_50_ was not significantly different to that of the other organs, overlapping the vulnerabilities of leaves and peduncles (*P* < 0.05).

**Fig. 2.**
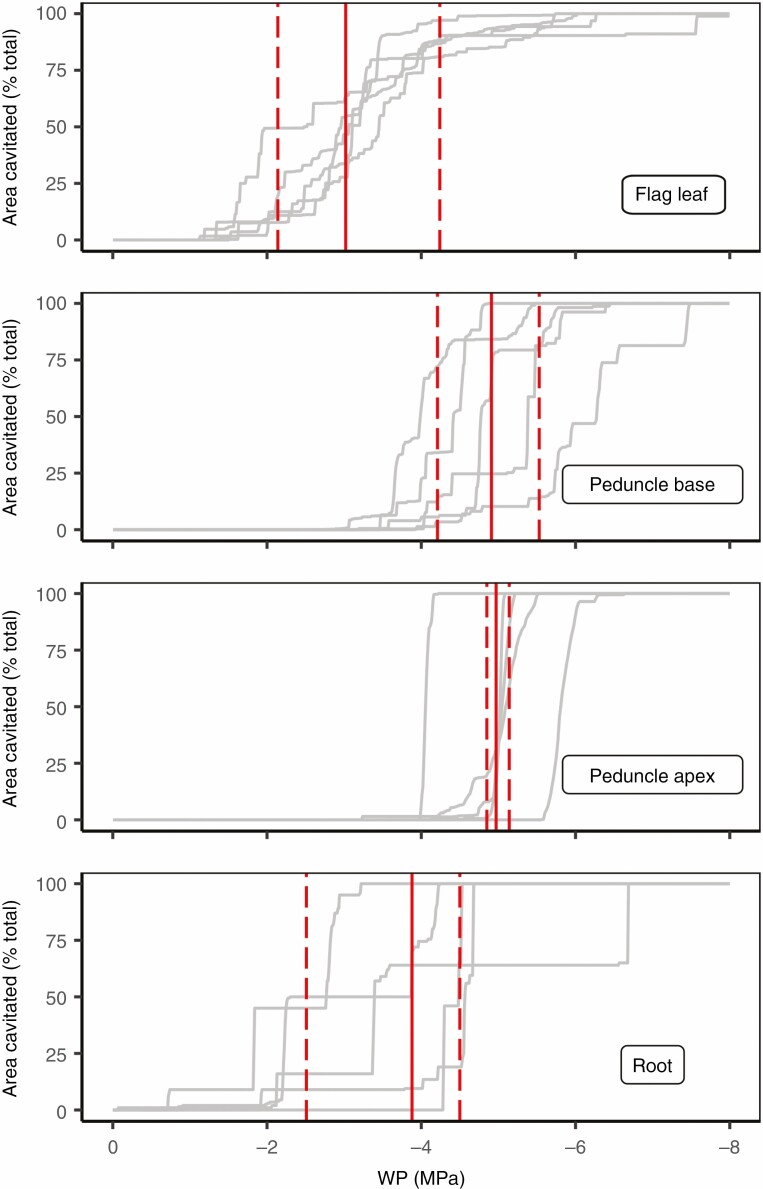
Vulnerability curves of five bread wheat plants showing cumulative percentage xylem cavitation in roots, flag leaves, and basal and apical regions of peduncles. Grey lines designate individual vulnerability curves for each replicate plant. Red vertical line indicates P_50_, and dashed vertical lines show the P_12_*–*P_88_ margins for each organ. Each root vulnerability curve (grey) represents one individual plant containing two to six roots.

The individual vulnerability curves revealed differences in the pattern and timing of cavitation between organs and amongst replicates (Fig. 2). The leaf vulnerability curves were very similar in shape across replicate plants, with most cavitation occurring early and a few final embolisms skewing the curve to show a P_88_ more negative than −4 MPa. Cavitation throughout the peduncle generally only started as cavitation was finishing in the leaves. Once initiated, cavitation in peduncles proceeded rapidly, as indicated by the similarity of P_12_ and P_88_, with all the vessels in these organs cavitating in quick succession. This was particularly apparent at the peduncle apex. The two to six roots per plant were grouped together to produce one root vulnerability curve to examine the differences between replicate plants (with the cavitated pixel values of each root segment of the bundle contributing to the vulnerability curve per plant) (Fig. 2). Large variation in the shapes of these grouped root vulnerability curves were observed between replicate plants (Fig. 2), contributing to the large s.d. around the *P*_50_ (Fig. 1). These root vulnerability curves began at very mild water stress and continued until very negative WP values.

**Fig. 3.**
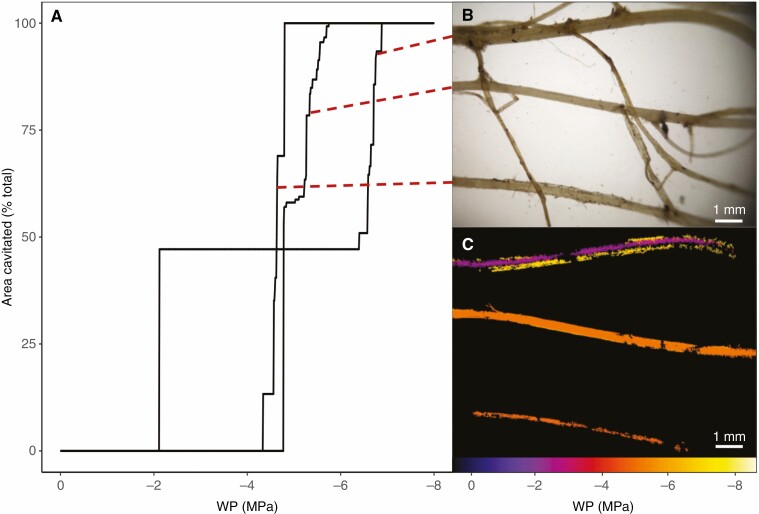
Vulnerability to cavitation of three large roots within a single example replicate plant. (A) One vulnerability curve for each of the large roots sampled in the plant. Each black line indicates cumulative cavitation events within an individual large root. (B) The corresponding roots (indicated by the dashed red line), and (C) the colour-mapped water potential (WP) causing cavitation in each root.

Due to the large observed variation in the root vulnerability between individual replicate plants, and large deviation around the *P*_50_ mean (Figs 1 and 2), the vulnerability curves of multiple root segments within a plant were examined separately (one unique curve from the cavitated pixels of each root segment) and found to be highly variable even within a single plant (Fig. 3).

**Fig. 4.**
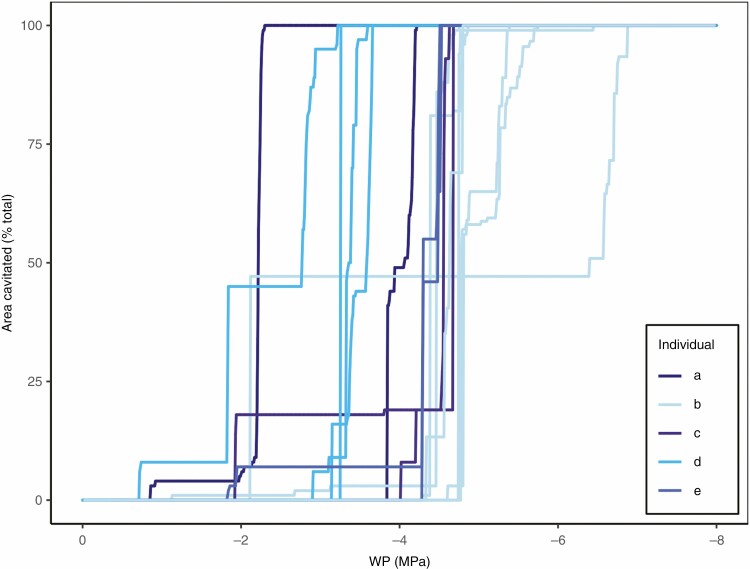
Vulnerability curves of all measured roots across five replicates of bread wheat (one unique vulnerability curve produced for each root). Each line indicates cavitation within one large root. Colour designates the two to six large roots within one replicate individual plant.

When these vulnerability curves for each root within a plant were examined across replicates (resulting in 16 individual root vulnerability curves), even more variation was observed (Fig. 4). Although the individual root segments often showed steep vulnerability curves, the WP triggering cavitation was highly variable among root segments of a single plant and between plants (Fig. 4). Although many root vulnerabilities were clustered around the root mean *P*_50_ of −4.05 MPa, this varied extensively across the roots measured, and among the —two to six root segments per individual at least one remained uncavitated at below −4 MPa (Fig. 4).

### Post-stress rehydration

**Fig. 5.**
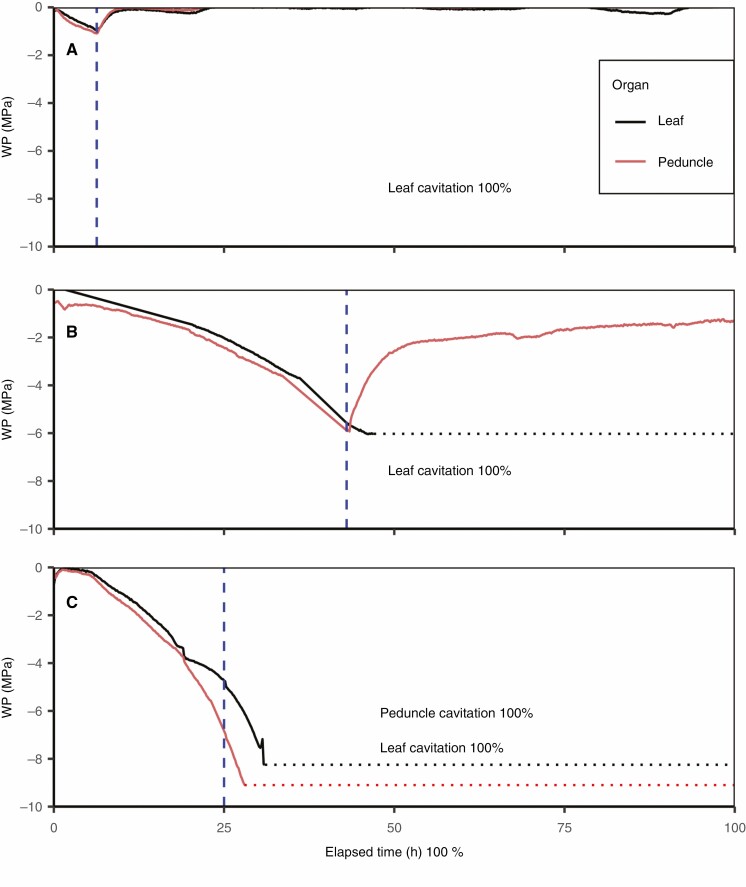
Recovery of wheat flag leaf and peduncle when rehydrated after increasing water stress. Plant A was dried to limited water stress with no leaf cavitation. Plant B was dried to the point of 100 % leaf cavitation and then rewatered to confirm the functionality of the water supply in the peduncle and roots. Plant C was dried beyond the expected peduncle P_100_ water potential and then rewatered. Dashed blue lines indicate the time of rewatering. Dashed red/black lines indicate the point when runaway cavitation in each organ resulted in the psychrometer being unable to achieve a reliable Peltier cooling curve (see Supplementary Data Fig. S2 for raw data).

Plants dried to water stress severe enough to cause major cavitation in the flag leaves showed rehydration of the peduncles and thus roots but not leaves upon rewatering (Fig. 5). When dried to −1 MPa, before leaf cavitation, and rewatered, the plant fully rehydrated within 3 h (Fig. 5A). When dried to the point of 100 % leaf cavitation (Fig. 5B), the peduncle rehydrated slowly and leaves failed to rehydrate. When pushed beyond the expected WP threshold of peduncle P_100_, both the leaves and peduncles failed to rehydrate, showing that peduncle hydration was contingent on an undamaged xylem connection (Fig. 5C). Additional replicates can be found in Supplementary Data Fig. S3.

## DISCUSSION

The xylem in the roots and peduncles of wheat plants showed greater resistance to water stress-induced cavitation than the leaves, supporting our hypothesis that water supply to reproductive tissues would be maintained during extreme drought thus maximizing the chance of reproductive success. The functional implications of building greater cavitation resistance in tissues supplying the seeds compared with leaves were demonstrated during rehydration. Xylem supplying the wheat spike maintained sufficient capacity to rehydrate the reproductive tissue after a drought stress strong enough to completely cavitate and kill the flag leaf.

While we have examined the ecophysiological consequences of drought in a commercial wheat variety with many potential selected traits, the reproductive strategy and morphology of wheat are similar to many grasses (Volaire, 2003; Norton *et al.*, 2016). We investigated wheat during a narrow window of its life cycle, but the part we have chosen to examine (during the essential grain filling stage) has particular relevance to the reproductive success of the plant (Farooq *et al.*, 2014; Zeiter *et al.*, 2016). Under the conditions used here, the observed priority of water supply through roots to reproductive tissues would be expected to increase fitness during adverse, but not uncommon, conditions.

Theoretically, according to our measured distribution of xylem vulnerabilities, when rewatered at WP values dry enough to cavitate the leaves, the root and peduncles should have maintained enough functionality to support a rehydrating water supply to the seed. Our rehydration experiments confirmed that the theoretical segmented vulnerability data corresponded to an observable recovery in resistant tissues (stems supplying flowers) and damage in more vulnerable tissues (leaves). Thus, water supply to the seed could be recovered even after the early sacrifice of evaporatively costly leaves, revealing the potential for reproductive completion after drought. This reproductive water supply bias is consistent with findings for domesticated tomato, reflecting the necessity in annual plants to reproduce quickly even under water stress (Harrison Day *et al.*, 2021). The pattern observed here contrasts strongly with vulnerability segmentation patterns observed in perennial plants which were found to sacrifice reproductive water supply early during drought (Zhang and Brodribb, 2017; Bourbia *et al.*, 2020). This difference in vulnerability traits reflects the contrasting strategy of the different growth habits. In woody plants which require significant carbon resources to grow, it is necessary to build plants that will survive successive droughts over multiple seasons (Sperry and Love, 2015). In contrast, annual grasses will probably face only one water deficit during their shorter life cycles, with the priority of water supply dedicated to seed-set over short-lived leaves that will senesce at the end of the season anyway. These segmented vulnerability traits in annual and perennial plants probably contribute to a continuum of drought avoidance and resistance strategies, associated with varying growth habits, that range from flower and leaf senescence to seasonal dormancy during dry periods (Volaire and Norton, 2006; Norton *et al.*, 2009; Volaire *et al.*, 2009). In the wheat plants here, our rehydration experiments support the concept of reproductive priority under drought stress in annual plants.

Our analysis of root vulnerability presents new insight into the hydraulic traits of wheat, providing key context linking the aerial vulnerability traits with the water relationships below ground. Given the importance of the roots for water uptake, examining root vulnerability has key relevance for understanding plant hydraulics and recovery after drought. Roots were found to be on average more cavitation-resistant than leaves but showed a large range in vulnerabilities within and between plants. Studies of xylem cavitation in woody plant roots typically involve excision of pieces of the root to use hydraulic flow methods such as air-injection, centrifuge and bench dehydration (Sperry and Saliendra, 1994; Alder *et al.*, 1997; Sperry and Ikeda, 1997; Kavanagh *et al.*, 1999; Pratt *et al.*, 2015, Johnson *et al.*, 2016). These methods suggest that roots are more vulnerable to cavitation than trunks and branches. By contrast, non-invasive visual methods such as the OV technique and micro-CT suggest roots to be similar in vulnerability or tougher than leaves (Skelton *et al.*, 2017; Rodriguez-Dominguez *et al.*, 2018). Measures of cavitation vulnerability in the roots of herbaceous plants and grasses have used various methods to assess embolism in excised roots (Cochard *et al.*, 1994; Alder *et al.*, 1996; Buchard *et al.*, 1999; Cochard, 2002; Kaufmann *et al.*, 2009), but our data provide the first detailed non-invasive examination of the timing and variation in root cavitation in an annual species in parallel with above-ground tissues.

The extensive variation found in xylem vulnerability between multiple roots of an individual, and in the whole-root character between plants, was unexpected. Across the similarly sized roots of all the replicates, the WP causing 100 % cavitation in a single root segment varied between −2 and −6 MPa. This suggests that a proportion of the root bundle in each individual cavitates very early in drought, while a small number of roots remain functional for much longer, providing the potential to supply water transport if rain occurs after drought. This variation also highlights that describing root vulnerability as a mean value does not fully explain the hydraulic behaviour of the root systems, and a closer examination of the individual root variation and breadth of vulnerability curves is necessary to understand these hydraulic relationships below ground. In this picture of hydraulic vulnerability segmentation, the roots supply the key context for whole-plant hydraulic dynamics, explaining how water supply may be recovered after drought severe enough to kill the leaves through a small percentage of resistant roots. While this enduring root conductance explains the rehydration of the peduncle following drought, root refilling may also provide another explanation for the recovery of root function, and deeper study of intact root systems in soil is necessary to comprehensively understand the dynamics of whole root networks (McCully *et al.*, 1998; Buchard *et al.*, 1999; McCully, 1999; Schenk *et al.*, 2021).

In these wheat plants, the pathway of water supply in drought is finely linked to the growth-habit and ecological drivers of this annual grass species, with investment in cavitation-resistant xylem functionally supporting reproductive opportunity under unfavourable conditions. Here, the segmented xylem vulnerabilities within the plant provide insight into the hydraulic strategies of these plants under drought conditions, optimizing the reproductive fitness of the plants under stress. These hydraulic traits reveal key resistances and vulnerabilities in the pathway of water through the highly variable root networks to the early sacrificed leaves and cavitation-resistant reproductive tissues. Given the overlap in the vulnerability to cavitation of a few final roots with the peduncles, the hydraulic rescue of reproductive tissue in wheat after severe drought stress appears contingent on a small percentage of tough roots. The segmented hydraulic vulnerabilities within these wheat plants highlight that tissue cavitation must be examined from a whole plant perspective to understand the broader physiological patterns and timing of damage under acute water stress.

## Supplementary Material

mcad048_suppl_Supplementary_Material

## SUPPLEMENTARY DATA

**Fig. S1**: Additional replicates of rehydration tests in plants dried to increasing water stress. **Fig. S2**: Example showing the rate of WP decline in a leaf and peduncle of an example plant. **Fig. S3**: Supplementary additional replicates of Fig. 5.
