# Supplementary material for: Resistant xylem from roots to peduncles sustains reproductive water supply after drought-induced cavitation of wheat leaves
Source: Ann Bot. 2023 Mar 22;131(5):839–50. doi: 10.1093/aob/mcad048 (PMC10184455; doi:10.1093/aob/mcad048)
Supplement: mcad048_suppl_Supplementary_Material [file mcad048_suppl_supplementary_material.doc]

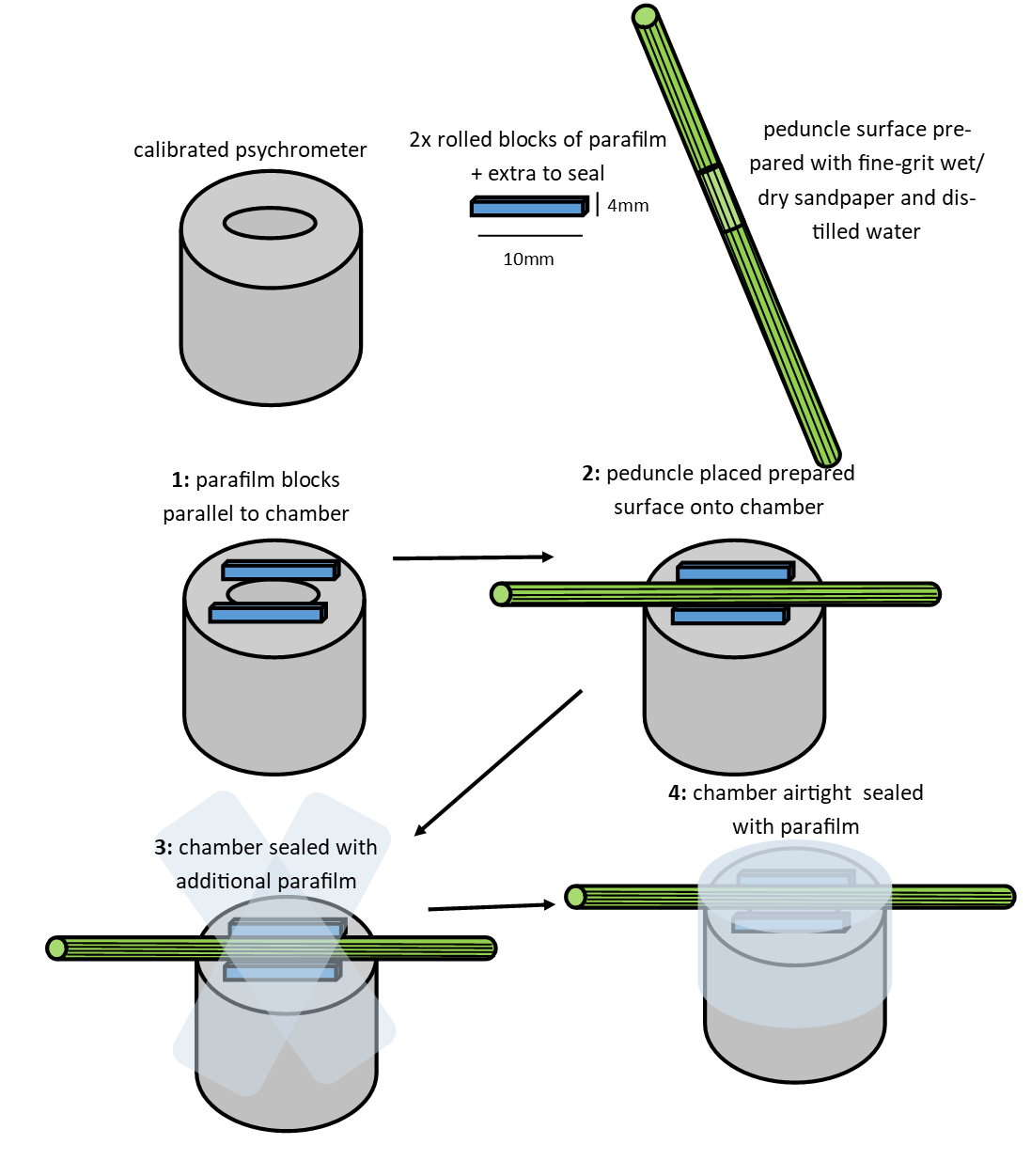


**Figure S1**: Procedure for placing stem psychrometer on the peduncle of wheat.


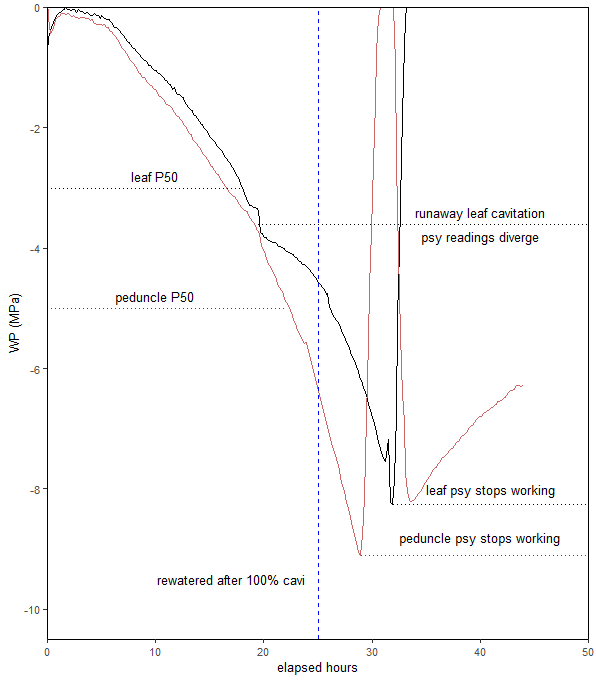


**Figure S2**: Example figure (taken from subset G in figure S1) showing the rate of WP decline in a leaf and peduncle of an example plant. Leaf and peduncle rates of dehydration remain closely associated until the point of runaway leaf cavitation (after leaf P50). Both psychrometers stop working when the dry tissue is unable to sustain a reliable Peltier cooling curve, resulting in unreliable psychrometer data after ~28 elapsed hours in this example.

**
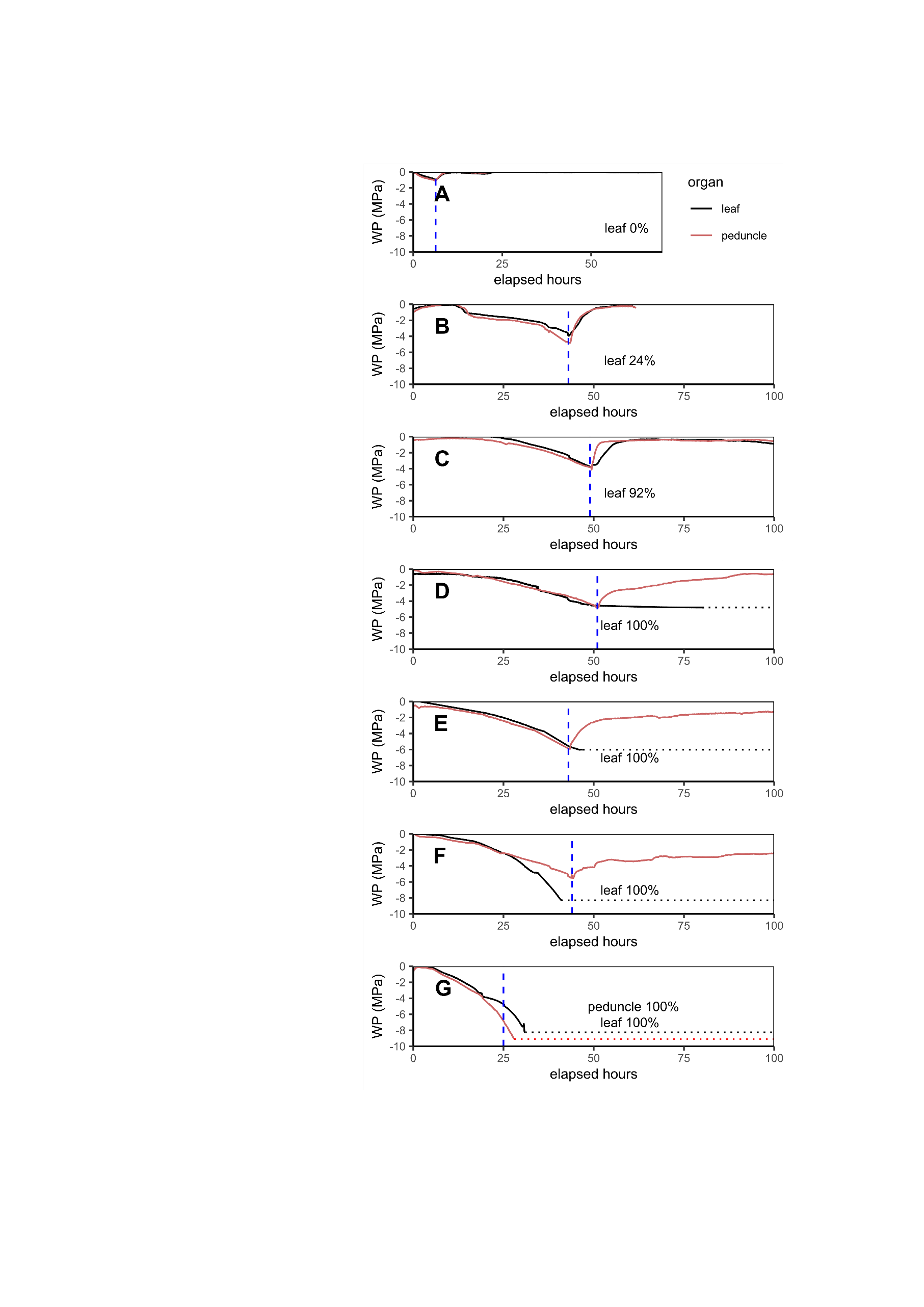
**

**Figure S3**: Supplementary additional replicates of figure 5. Recovery of wheat flag leaf and peduncle when rehydrated after increasing water stress, and the corresponding leaf cavitation. Facets A, F and G are shown in figure 5. Dashed blue lines indicate the time of rewatering, dashed red/black lines indicate when organs are fully cavitated and psychrometers stop reading. *Dehydration of leaf in facet D calculated from the shrinkage and swelling of leaf diameter in the absence of paired leaf and peduncle psychrometer data, see (Bourbia et al., 2021, Bourbia et al., 2020).

**Literature cited:**

**Bourbia I, Carins‐Murphy MR, Gracie A, Brodribb TJ.** **2020**. Xylem cavitation isolates leaky flowers during water stress in pyrethrum. *New Phytologist,* **227**: 146-155.

**Bourbia I, Pritzkow C, Brodribb TJ.** **2021**. Herb and conifer roots show similar high sensitivity to water deficit. *Plant Physiology,* **186**: 1908-1918.
